# Left ventricular ejection fraction decrease related to BRAF and/or MEK inhibitors in metastatic melanoma patients: A retrospective analysis

**DOI:** 10.1002/cam4.2922

**Published:** 2020-02-14

**Authors:** Mathilde Berger, Mona Amini‐Adlé, Delphine Maucort‐Boulch, Philip Robinson, Luc Thomas, Stéphane Dalle, Pierre‐Yves Courand

**Affiliations:** ^1^ Service de dermatologie Centre Hospitalier Lyon Sud Hospices Civils de Lyon Pierre‐Bénite France; ^2^ Service de Biostatistiques Hospices Civils de Lyon Université Lyon 1 Lyon France; ^3^ Laboratoire de Biométrie et Biologie Evolutive Université de Lyon CNRS Equipe Biostatistique‐Santé Villeurbanne France; ^4^ Direction de la Recherche Clinique et de l’Innovation Hospices Civils de Lyon Lyon France; ^5^ Centre de Recherche en Cancérologie de Lyon Université de Lyon Université Claude Bernard Lyon France; ^6^ Hospices Civils de Lyon Service de cardiologie Hôpital de la Croix‐Rousse et Centre Hospitalier Lyon Sud Lyon France; ^7^ Université de Lyon Creatis Umr Inserm U1044 INSA Lyon France

**Keywords:** adverse events, BRAF inhibitor, cardiac toxicity, heart failure, left ventricular ejection fraction, left ventricular systolic dysfunction, MEK inhibitor, metastatic melanoma

## Abstract

BRAF and MEKis have revolutionized the management of BRAF^V600^‐mutated melanoma patients. Left ventricular ejection fraction decrease (LVEF‐D) related to these treatments has not been thoroughly evaluated to date. The main objective of this study was to describe characteristics of LVEF‐D in melanoma patients treated with BRAF and/or MEKis. Metastatic melanoma patients treated with BRAF and/or MEKis between March 1, 2012 and May 18, 2018 were included retrospectively (Lyon Sud University Hospital, Hospices Civils de Lyon). LVEF‐D was defined as a reduction in LVEF ≥10% from baseline to a value <55%; normalization was defined as a value ≥55%. Among the 88 patients included, 12 (13.6%) experienced a LVEF‐D, including 10 grade 2 and 2 grade 3. The median onset of which was 11 months (IQR [3‐21]). No patient previously treated with beta‐blockers (n = 12) experienced a LVEF‐D. Analysis of laboratory parameters, electrocardiogram, and transthoracic echocardiography during the follow‐up did not find any predictive marker of LVEF‐D. All patients who benefited from a specific treatment of LVEF‐D had a normalization of LVEF at the end of follow‐up. LVEF recovery was significantly better for patients treated with angiotensin converting enzyme inhibitors and beta‐blockers than those who did not (*P* = .019). Ophthalmological adverse events were significantly more frequent in patients who experienced a LVEF‐D (*P* = .006) and the latter did not influence overall‐survival (*P* = .117) or progression‐free‐survival (*P* = .297). LVEF‐D is a common and easily manageable adverse event due to BRAF and MEKis. Its association with ocular toxicity suggests a close ophthalmological monitoring when LVEF‐D occurs.

## Graphical abstract

Left ventricular ejection fraction decreased was observed in 13.6% of patients treated with BRAF and MEK inhibitors. All patients treated with beta‐blockers and/or angiotensin converting enzyme inhibitors had a normalization of left ventricular ejection fraction. Half of patients with left ventricular ejection fraction decreased had also ophthalmologic adverse event.

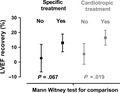

## 1 INTRODUCTION

Cutaneous melanoma is the most lethal skin cancer, and its incidence continues to increase worldwide. Patients with metastatic melanoma have a poor prognosis, with a 5‐year‐survival rate below 25%.^1^ Recently, the development of targeted therapies with BRAF and MEKis (BRAF and MEK inhibitors) has revolutionized the management and the prognosis of BRAF^V600^‐mutated melanoma patients. 3 combinations of BRAF and MEKis are currently approved by the Food and Drug Administration and used for the treatment of advanced BRAF^V600^‐mutated melanoma: vemurafenib‐cobimetinib,^2^ dabrafenib‐trametinib,^3^, ^4^ and, more recently, encorafenib‐binimetinib.^5^ A new spectrum of side effects has emerged related to these treatments, including several types of cardiovascular adverse events (AEs) such as left ventricular ejection fraction decrease (LVEF‐D), QT interval prolongation, hypertension, and peripheral edema.^6^, ^7^ LVEF‐D induced by BRAF and MEKis, although widely reported in clinical trials, has never been thoroughly described to date and data regarding its management are limited.^8^, ^9^, ^10^ The main objective of the study presented herein was therefore to describe the characteristics of LVEF‐D in a large cohort of metastatic melanoma patients treated with BRAF and/or MEKis in a real‐life setting. Secondary objectives were to determine whether laboratory, electrocardiogram (ECG) or transthoracic echocardiography (TTE) markers could identify sub‐clinical cardiac damage and be predictive of a LVEF‐D. Subsequent objectives were to describe the different types of management of LVEF‐D. In addition, whether or not other cardiovascular or extra‐cardiovascular toxicities were preferentially associated with LVEF‐D, and the impact of LVEF‐D on overall‐survival (OS) and progression‐free‐survival (PFS) were also investigated.

## 2 PATIENTS AND METHODS

### 2.1 Study population

We conducted a longitudinal retrospective observational single‐center study in a French university referral hospital (Centre Hospitalier Lyon Sud, Hospices Civils de Lyon). Patients with metastatic melanoma treated with BRAFi alone, MEKi alone, or combination therapy with BRAF and MEKis between March 1, 2012 and May 18, 2018 were included. Patients who did not receive regular TTE under treatment were excluded. The present study was approved by the regional ethics committee n°19‐121(Comité d’Éthique du Centre Hospitalier Universitaire de Lyon).

### 2.2 Patient follow‐up and data collection

For each patient, the follow‐up period started at the BRAFi initiation visit, or, for patients treated with MEKi alone this started at the MEKi introduction visit. Baseline data including age, sex, previous cardiovascular history, cardiovascular risk factors, current cardiovascular treatments, tumor burden (less or more than 3 metastatic sites), previous lines of treatment for melanoma, laboratory assessment, baseline ECG and TTE were retrospectively collected during this visit. End of follow‐up was the last visit before BRAFi discontinuation or at the last visit before death. Patients were censored on December 31, 2018.

### 2.3 Cardiac assessment

LVEF was determined by TTE, routinely performed before introduction of BRAF and MEKis, at 1 month, at three months, and every three months thereafter as recommended.^8^, ^11^ LVEF was assessed using Simpson's biplane method if feasible, otherwise using visual method. LVEF‐D was defined as a reduction in LVEF ≥10% from baseline to a value <55% according to the Cardiac Review and Evaluation Committee.^12^ Normalization was defined as a value ≥55%. LVEF‐D was graded according to the guidelines for management established by Welsh *et al*,^9^ and considered severe if grade ≥3. Specific treatment of LVEF‐D corresponded to the discontinuation of BRAF and/or MEKis ± angiotensin converting enzyme inhibitors (ACEi) and beta‐blockers introduction. Cardiotropic treatments refer to ACEi and beta‐blockers introduction. Diastolic function was evaluated using 4 criteria derived from recent guidelines: 1) average E/e’ >14, 2) Septal e’ velocity <7 cm/s or lateral e’ velocity <10 cm/s, 3) tricuspid regurgitation velocity >2.8 m/s, 4) left atrial surface >20 cm^2^ instead of left atrial volume index >34 mL/m^2^ which was not recommended during the enrollment period. Diastolic dysfunction was defined as the presence of ≥3 criteria.^13^ Valvular heart disease were described as recommended and considered significant if grade ≥2.^14^TTE data were retrospectively analyzed during 5 visits for patients with LVEF‐D: at baseline, last visit before LVEF‐D, visit with LVEF‐D, next visit after LVEF‐D, and at the end of follow‐up. In the subgroup of patients without LVEF‐D (as defined above), TTE data were analyzed only at baseline and at the last visit of follow‐up.

A standard 12‐lead ECG was recorded at 25 mm/s and 1 mV/cm at each follow‐up visit (every 4 weeks). ECG were interpreted blinded to clinical and laboratory parameters in the same way as TTE according to the presence or absence of LVEF‐D. The automatically measured heart rate, QRS duration, and PQ intervals were collected. QT interval was measured manually in each lead from the onset of the QRS complex to the end of the T wave. If the T wave and U wave were superimposed or could not be separated, the downslope of the T wave was extended by drawing a tangent on the steepest proportion of the downslope until it crosses the TP segment. The longest QT interval manually measured was then corrected for heart rate using Fridericia (QT corrected = QT/√^3^RR) and Bazett (QT corrected = QT/√RR) formulas with QT and RR in milliseconds and heart rate in beats/minute.^15^ Presence of electrical left ventricular hypertrophy (LVH) was assessed according two electrical criteria: R wave in aVL lead and Cornell voltage criterion (RaVL + SV3). Sokolow‐Lyon index was not performed because of its low sensitivity.^16^

### 2.4 Laboratory assessment

Laboratory assessment was routinely performed before introduction of BRAF and/or MEKis and at each visit of follow‐up (every 4 weeks). The values of serum potassium, serum calcium, creatinine, and hemoglobin were noted at baseline, and serum levels of creatine phosphokinase (CPK) were analyzed at baseline and at each follow‐up visit.

### 2.5 Adverse events

Occurrence of AEs was routinely assessed at each follow‐up visit (every 4 weeks) and graded according to the Common Terminology Criteria for Adverse Event (CTCAE) version 5.0. Occurrence of AEs was retrospectively analyzed at each follow‐up visit from the first visit following introduction of BRAFi (or MEKi for patients initially treated with MEKi alone) until 30 days after the end of BRAFi.

### 2.6 Statistical analysis

Variables were summarized as mean ± standard deviation (SD), or median [interquartile range, IQR] according to distribution. Categorical variables were expressed as numbers and percentages. Mann‐Whitney/Wilcoxon or Friedman tests were used to compare continuous variables for comparisons and longitudinal measures, respectively. The χ^2^ or Fisher's exact tests were used for comparisons of categorical variables. Kaplan‐Meier analysis with the Log‐rank test was used to estimate OS and PFS. All analyses were performed using the R software (R Core Team (2014). R: A language and environment for statistical computing. R Foundation for Statistical Computing, Vienna, Austria. URL http://www.R-project.org/). No correction was applied for multiple testing since these were considered as exploratory analyses. Comparisons of small subgroups without meaningful clinical hypotheses were not performed. A *P* value < .05 was considered significant.

## 3 RESULTS

### 3.1 Baseline characteristics of study population

A total of 88 patients were included (Figure S1). Among these, 11 patients (12.5%) had an overt cardiovascular disease and 28 patients (31.8%) cumulated ≥2 cardiovascular risk factors. A total of 18 patients (20.5%) were treated with BRAFi alone, including 2 patients who received monotherapy with encorafenib in a clinical trial. One patient included in a clinical trial received a MEKi alone (binimetinib). No patient was treated with the combination of encorafenib‐binimetinib. The median duration of treatment was 9 months (IQR [5‐20]). 30 patients (34.1%) had a rechallenge after progressive disease under previous treatment with BRAF and/or MEKis. There were 21 patients (23.9%) who had received previous immunotherapy, including 4 patients who received immunotherapy as adjuvant treatment for stage III melanoma in clinical trials (Table 1).

**Table 1:** Characteristics of study population

	Total (n = 88)	LVEF decrease (n = 12)	No LVEF decrease (n = 76)	*P* values
Demographic characteristics				
Age (years)	54.0 ± 16.2	51.6 ± 15.7	54.4 ± 16.3	.564
Men, n (%)	51 (58.0)	9 (75.0)	42 (55.3)	.331
Cardiovascular disease, n (%)	11 (12.5)	1 (8.3)	10 (13.2)	1
Coronary artery disease	5 (5.7)	0	5 (6.6)	
Stroke	3 (3.4)	0	3 (3.9)	
Peripheral artery disease	1 (1.1)	1 (8.3)	0	
Atrial fibrillation	2 (2.3)	0	2 (2.6)	
Cardiovascular risk factors ≥2, n (%)	28 (31.8)	5 (41.7)	23 (30.3)	.509
Hypertension	26 (29.5)	4 (33.3)	22 (28.9)	
Diabetes	6 (6.8)	2 (16.7)	4 (5.3)	
Dyslipidemia	7 (8.0)	2 (16.7)	5 (6.6)	
Current smoking	18 (20.9)	4 (33.3)	14 (18.9)	
BMI (kg.m^‐2^)	26.0 ± 5	26.7 ± 4.5	25.9 ± 5.1	
Cardiovascular treatment, n (%)	33 (37.5)	2 (16.7)	31 (40.8)	
ACEi or ARBs	18 (20.5)	2 (16.7)	16 (21.1)	1
Beta‐blockers	12 (13.6)	0	12 (15.8)	.206
Calcium channels blockers	11 (12.5)	1 (8.3)	10 (13.2)	
Diuretics	9 (10.2)	1 (8.3)	8 (10.5)	
Anti‐aldosterone	3 (3.4)	0	3 (3.9)	
Alpha‐blockers	2 (2.3)	0	2 (2.6)	
Statins, n (%)	12 (13.6)	1 (8.3)	11 (14.5)	1
Systolic blood pressure (mmHg)	129 ± 17	128 ± 20	129 ± 17	
Diastolic blood pressure (mmHg)	78 ± 12	79 ± 17	78 ± 11	
High tumor burden (> 3 metastatic sites), n (%)	28 (31.8)	5 (41.7)	23 (30.3)	.509
Melanoma treatment, n (%)				
BRAFi alone	18 (20.5)	3 (25.0)	15 (19.7)	
Vemurafenib alone	14 (15.9)	1 (8.3)	13 (17.1)	
Dabrafenib alone	2 (2.3)	1 (8.3)	1 (1.3)	
Encorafenib alone	2 (2.3)	1 (8.3)	1 (1.3)	
MEKi alone (binimetinib)	1 (1.1)	0	1 (1.3)	
Combination therapy	69 (78.4)	9 (75.0)	60 (78.9)	
Vemurafenib ‐ cobimetinib	40 (45.4)	4 (33.3)	36 (47.3)	
Dabrafenib ‐ trametinib	29 (33.0)	5 (41.7)	24 (31.6)	
Rechallenge, n (%)	30 (34.1)	5 (41.7)	25 (32.9)	
Duration of treatment (months)	9 [5‐20]	19 [11‐23]	8 [5‐18]	
Duration of rechallenge (months)	5 [3‐8]	4 [3‐5]	5 [3‐10]	
First‐line treatment, n (%)	71 (80.7)	10 (83.3)	61 (80.3)	
Previous immunotherapy, n (%)	21 (23.9)	2 (16.7)	19 (25.0)	.723
Anti‐PD1	13 (14.8)	1 (8.3)	5 (6.6)	
Anti‐CTLA4	2 (2.3)	0	2 (2.6)	
Combination	6 (6.8)	1 (8.3)	5 (6.6)	

Note

Unless otherwise stated, the data are given as means ± SD or medians [interquartile ranges].

Wilcoxon tests were used to compare continuous variables for unpaired comparisons and the χ^2^ or Fisher's exact tests were used for comparisons of categorical variables.

Abbreviations: ACEi, Angiotensin‐converting enzyme inhibitors; ARB, Angiotensin II receptor blockers; BMI, Body Mass Index.

Laboratory parameters were normal at baseline, including normal CPK level in all patients. Baseline ECG found LVH in 3 patients, 1st atrioventricular block in 2 patients, complete bundle branch block in 4 patients, and repolarization disorders in 2 patients. Similarly, LVEF was normal at baseline; the mean ± SD LVEF at 65.6% ± 5.0%. No patient had a diastolic dysfunction at baseline. Significant valvular heart disease was reported in 2 patients, and 3 patients had pericardial effusion (Table S1).

### 3.2 Comparison according to the presence or absence of LVEF‐D

No clinical, laboratory, electrocardiographic or echocardiographic parameter was significantly different at baseline between patients who experienced LVEF‐D (reduction in LVEF ≥10% from baseline to a value <55%; n = 12, 13.6%) and those who did not. No patient previously treated with beta‐blockers experienced LVEF‐D under BRAF and/or MEKis (*P* = .206). There was no significant difference in the frequency of previous immunotherapy treatment between those with and without LVEF‐D (Table 1; Table S1).

### 3.3 Changes of echocardiographic, ECG, and laboratory parameters during treatment with BRAF and/or MEKis

Among the 12 patients who had LVEF‐D, 10 patients had an asymptomatic grade 2 and 2 patients had a symptomatic grade 3 LVEF‐D; no patient presented grade 4 LVEF‐D. The median decrease of LVEF was 15.5% (IQR [13.2‐17.4]), and the median time to onset was 11 months (IQR [3‐21]). No patient (0/19) treated with BRAFi or MEKi alone experienced a LVEF‐D *vs*. 17.4% of patients (12/69) treated with combination therapy. LVEF‐D occurred during the initial treatment period for 10 patients and during the rechallenge period for 2 patients. Among the 3 patients who presented a LVEF‐D during initial treatment and who were then rechallenged, none experienced recurrence of LVEF‐D during the rechallenge period. There were no clinical meaningful variations of heart rate, PR interval, QRS interval, QT corrected interval, repolarization disorders, and LVH indexes during follow‐up (Table 2).

**Table 2:** Variations of electrocardiographic and echocardiographic parameters during treatment with BRAF and/or MEK inhibitors for patients who had LVEF decrease

	Baseline	Last visit before LVEF decrease	Visit with LVEF decrease	Next visit after LVEF decrease	End of follow‐up	*P* value
Electrocardiographic parameters	n = 12	n = 12	n = 12	n = 10	n = 10	
Heart rate (bpm)	72 ± 10	79 ± 14	71 ± 16	63 ± 10	72 ± 10	
PR interval (ms)	143 ± 28	144 ± 35	150 ± 35	151 ± 33	147 ± 32	
QRS duration (ms)	89 ± 16	95 ± 23	95 ± 29	98 ± 32	99 ± 30	
Repolarization disorders, n (%)	1 (8.3)	3 (27.3)	2 (18.2)	2 (22.2)	2 (16.7)	
QT interval (ms)	385 ± 35	367 ± 29	382 ± 37	391 ± 41	386 ± 44	
QTc (Bazett) (ms)	418 ± 25	417 ± 23	413 ± 36	399 ± 49	417 ± 49	
QTc (Fridericia) (ms)	407 ± 26	400 ± 19	402 ± 31	396 ± 44	406 ± 42	
Cornell (mm)	12.3 ± 6.1	17.7 ± 5.4	16 ± 7.9	17.1 ± 8.2	17.4 ± 8.9	
RaVL (mm)	4.2 ± 2.2	5.5 ± 1.8	5 ± 1.4	5.2 ± 1.7	4.9 ± 2.3	
Echocardiographic parameters	n = 12	n = 9	n = 12	n = 11	n = 12	
LVEF (%)	65.7 ± 5.0	60.7 ± 6.0	50.1 ± 5.0	56.7 ± 9.0	59.4 ± 6.0	.0045
E/A ratio	0.9 ± 0.3	0.9 ± 0.4	1.1 ± 0.4	1.4 ± 1.5	1 ± 0.3	
Deceleration time of E wave (ms)	213 ± 63	195 ± 59	189 ± 40	202 ± 42	200 ± 43	
E/E' ratio	5.9 ± 1.4	6.2 ± 3.1	6.8 ± 2.1	6.8 ± 3.4	6.4 ± 2.1	
Left atrium surface (cm^2^)	15.6 ± 2.8	17.3 ± 2.2	18.4 ± 2.4	16.7 ± 4.6	17.3 ± 3.3	
PASP (mmHg)	29.5 ± 4.7	31.5 ± 7.1	28.3 ± 8.4	24.4 ± 4.8	27.6 ± 4.6	

Note

Unless otherwise stated, the data are given as means ± SD.

Friedman tests were used to compare continuous variables for longitudinal measures.

Abbreviations: LVEF, Left ventricular ejection fraction; PASP, Pulmonary artery systolic pressure; QTc, Corrected QT interval.

**Figure 1:**
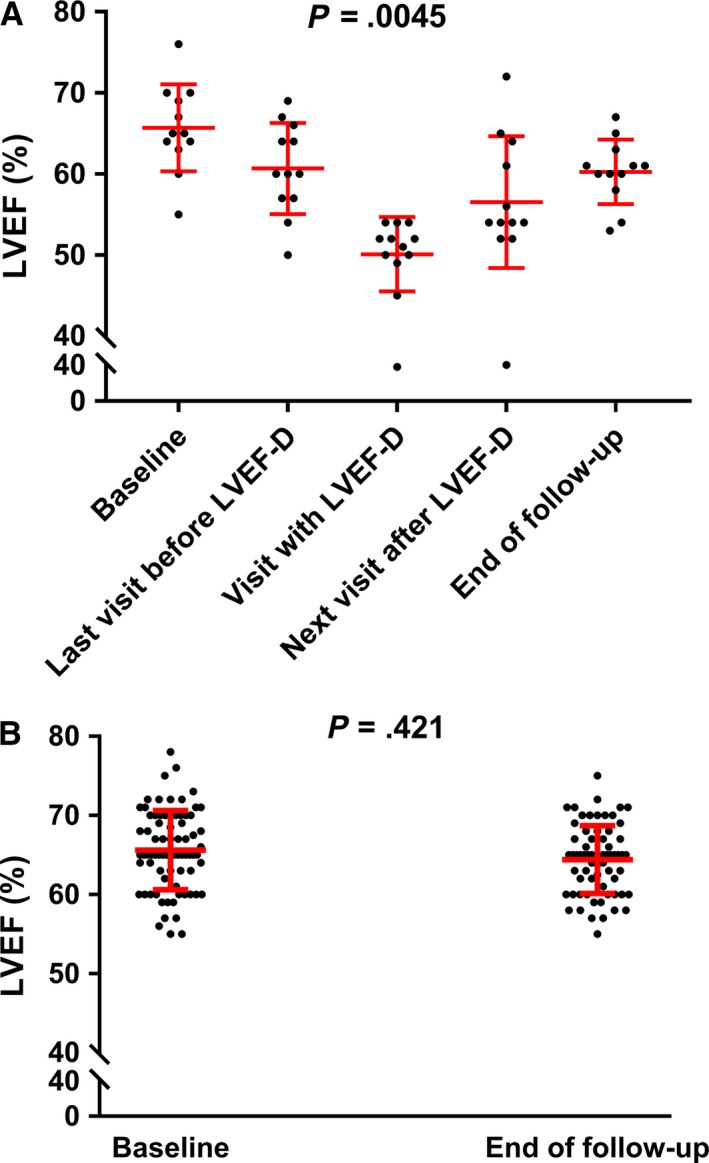
LVEF during treatment with BRAF and/or MEK inhibitors for patients who experienced LVEF decrease (A) and those who did not (B). The boxes show the interquartile range, with the median value indicated by the horizontal line; whiskers show the range

Patients with LVEF‐D had a significant variation of LVEF during follow‐up (*P* = .0045; Table 2); this was characterized by a progressive decrease from baseline to the lowest value of LVEF followed by a progressive recovery at the end of follow‐up (Figure 1A). Diastolic dysfunction was not observed before LVEF‐D (Table 2). Patients with LVEF‐D had a higher mean ± SD CPK level during follow‐up (673 ± 878 IU/L) than at baseline (50 ± 38 IU/L, *P* = .081; data available for n = 8).

Among patients without decrease of LVEF (Figure 1B), there was no marked difference in ECG and TTE parameters between baseline and the end of follow‐up (Table S2). There was a significantly higher mean ± SD CPK level under treatment with BRAF and/or MEKis (408 ± 507 IU/L) than at baseline (82 ± 105 IU/L, *P* < .001; data available for n = 57). No significant difference was observed between patients with or without LVEF‐D considering higher mean ± SD CPK level (673 ± 878 IU/L vs. 408 ± 507 IU/L, *P* = .629, respectively).

### 3.4 Management of patients with LVEF‐D

LVEF‐D management was not the same for all patients. Specific treatment (discontinuation of BRAF and/or MEKis ± ACEi and beta‐blockers introduction) was implemented in 8 patients, and 5 patients benefited from cardiotropic treatments with ACEi and beta‐blockers. No specific treatment has been implemented for 4 patients.

BRAF and MEKis were permanently discontinued in 1 patient due to grade 2 LVEF‐D associated with other grade 3 AEs. BRAF and/or MEKis were temporarily discontinued in 3 patients (Figure S2). The mean duration of interruption was 23 ± 7 days. No patient had recurrence of LVEF‐D after BRAF and/or MEKis reintroduction. Median PFS after LVEF‐D was 8 months (IQR [6‐9]) for patients whose BRAF and/or MEKis were discontinued, and 7 months (IQR [6‐16]) for the others.

**Figure 2:**
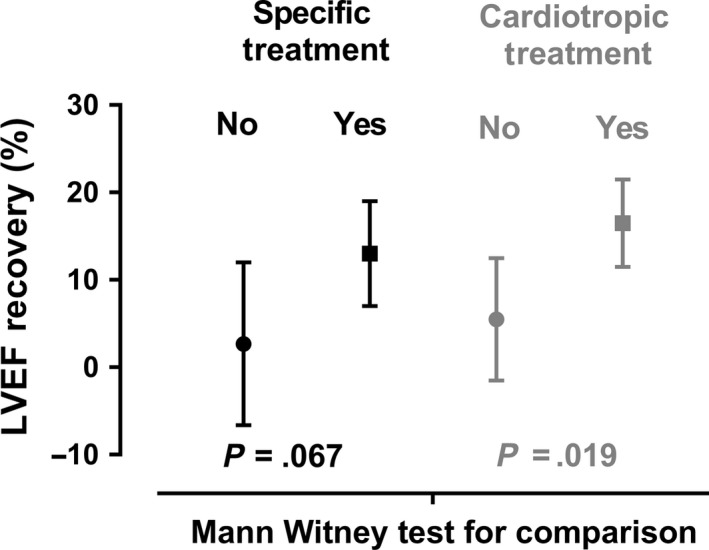
LVEF recovery according to the type of management. Circles and squares indicate the median value. Whiskers indicate standard deviation. The black square represents patients who received a specific treatment of LVEF decrease, whereas the black circle represents patients who did not. The grey square corresponds to patients who received cardiotropic treatments, whereas the grey circle represents patients who did not

All patients who benefited from a specific treatment had a normalization of LVEF at the end of follow‐up. The median time to LVEF normalization for these patients was 64 days (IQR [35‐76]). Among the 4 patients with no therapeutic change, MEKi was rapidly stopped in one patient due to another AE and no echocardiographic follow‐up was performed afterwards; the other 3 patients experienced LVEF normalization after a dose reduction of BRAF and MEKis due to other AEs. The median time to LVEF normalization for these patients was 97 days (IQR [62‐174]). At the end of follow‐up, the mean ± SD increase in LVEF after decrease was 13.0% ± 6.0% in case of specific treatment, versus 2.7% ± 9.3% for patients with no therapeutic change, with no significant difference between the two groups (*P* = .067). LVEF recovery was significantly better for patients who benefited from cardiotropic treatments (16.5% ± 5.0%) than those who did not (5.5% ± 7.0%, *P* = .019; Figure 2; Table 3).

**Table 3:** LVEF recovery according to the type of management

	Total (n = 12)	Specific treatment			Cardiotropic treatments		
		With (n = 8)	Without (n = 4)	*P* value	With (n = 8)	Without (n = 4)	*P* value
LVEF (%)							
At baseline	65.7 ± 5.4	66.1 ± 3.6	64.8 ± 8.6		66.2 ± 3.7	65.3 ± 6.5	
Visit with LVEF decrease	50.1 ± 4.6	48.7 ± 5.1	52.7 ± 1.5		47.4 ± 6.0	52.0 ± 2.1	
At the end of follow‐up	59.4 ± 6.1	61.3 ± 4.5	55.0 ± 7.9		62.8 ± 3.1	57.2 ± 6.7	
LVEF decrease from baseline to the lowest value	16.6 ± 5.1	17.4 ± 5.2	15.0 ± 5.3		18.8 ± 6.3	15.0 ± 3.8	
LVEF increase from the lowest value to the end of follow‐up	9.9 ± 8.3	13.0 ± 6.0	2.7 ± 9.3	.067	16.5 ± 5.0	5.5 ± 7.1	.019

Note

The data are presented as means ± SD.

Paired t‐tests were used to compare continuous variables before and after treatment.

### 3.5 Association between LVEF‐D and other AEs

Ophthalmologic AEs were significantly more frequent in patients who presented LVEF‐D (50.0%, n = 6) than those who did not (21.0%, n = 16, *P* = .006). There were 3 serous central retinopathy, 1 retinal pigment epithelial detachment, and 2 uveitis. Ophthalmologic AEs occurred before LVEF‐D in 3 patients, and after LVEF‐D in the other 3. Other cardiovascular and extra‐cardiovascular AEs are detailed in supplementary data (First paragraph, Table S3).

### 3.6 Overall‐survival (OS) and Progression‐free‐survival (PFS)

**Figure 3:**
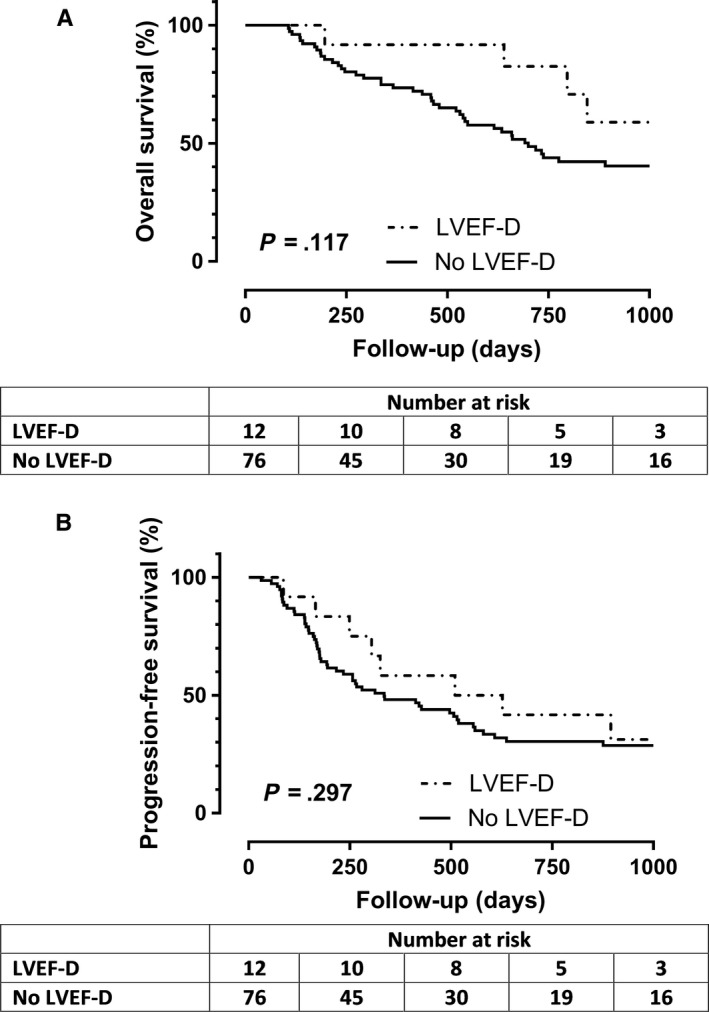
Kaplan‐Meier estimation of overall‐survival (A) and progression‐free‐survival (B) in patients who experienced LVEF decrease (LVEF‐D) and those who did not (no LVEF‐D)

OS and PFS were not significantly different between patients who presented LVEF‐D and those who did not (*P* = .117 and *P* = .297 respectively; Figure 3).

## 4 DISCUSSION

The present study found that LVEF‐D was common but usually not severe and had no significant impact on OS or PFS. None of the tested laboratory, ECG, or TTE parameters was found to be predictive of LVEF‐D, although ophthalmological AEs were significantly more frequent among those affected, and recovery was better in case of introduction of ACEi and beta‐blockers.

LVEF‐D was widely documented in clinical trials, reported in 0% to 12% of patients treated with BRAF ± MEKi,^2^, ^3^, ^4^, ^17^, ^18^, ^19^ which is slightly lower than that found herein. This difference can be explained by the absence of universal definition of LVEF‐D. Whereas in these clinical trials LVEF‐D was defined as a decline in LVEF ≥10% to final LVEF <50%. We chose 55% in order to be in agreement with the guidelines for the management of BRAF and MEKis.^8^, ^9^, ^10^ In the present study, 3 patients (3.4%) presented a decline in LVEF ≥10% to a value <50% (data not shown). This frequency is consistent with that reported in clinical trials.^2^, ^3^, ^4^, ^17^, ^18^, ^19^

To the best of our knowledge, the laboratory, ECG, and TTE parameters investigated herein as potentially predictive of LVEF‐D have not been investigated elsewhere. It is of note, however, that we could not analyze troponins, B‐type natriuretic peptide (BNP), or global systolic longitudinal strain (GLS) which have been found to be predictive of the occurrence and severity of LVEF‐D due to other cancer therapies.^20^, ^21^, ^22^, ^23^, ^24^, ^25^, ^26^, ^27^, ^28^ These parameters are not routinely measured in patients treated with BRAF and MEKis and therefore prospective studies may be conducted in the future to determine whether these parameters could predict cardiac dysfunction in the context of therapeutic BRAF and MEK inhibition.

In the present study, patients who experienced LVEF‐D did not all receive the same management. Although no recommendation mentions the possibility of using ACEi and beta‐blockers in the context of LVEF‐D induced by BRAF and MEKis, they were nevertheless introduced in some patients, and this is likely to have been by analogy with management of anthracyclines and other cancer therapies‐induced heart injury.^29^, ^30^, ^31^ It is of note that some results in the present study suggest a beneficial role of ACEi and beta‐blockers as LVEF recovery was significantly better when such cardiotropic treatments were introduced, and, although not significant, no patient previously treated with beta‐blockers experienced LVEF‐D under BRAF and MEKis. Furthermore, the effectiveness of beta‐blockers in such patients is biologically plausible; the myocardial beta‐adrenergic receptor activation signals through both the cardioprotective MEK/ERK axis and the cardiotoxic p38 MAP kinase pathway.^32^, ^33^ Theoretically, inhibiting MEK could shunt beta‐adrenergic signaling toward p38,^34^, ^35^ hence increasing the deleterious effects of MEK inhibition. Beta‐blockers might attenuate these effects directly by inhibiting beta‐adrenergic receptor‐mediated p38 activation. To the best of our knowledge, the mechanism of action of ACEi on cardiomyocytes specifically exposed to BRAF and MEKis has never been evaluated to date, but their beneficial role in cardiotoxicity due to other anticancer treatments is widely documented.^29^, ^30^, ^31^ Hence, a new management algorithm of LVEF‐D including use of ACEi and beta‐blockers may be proposed (Figure S3), as well as the possibility of using them as preventive treatments, but before implementation these require validation in a large prospective study.

The potential association between LVEF‐D and other toxicities has not been previously evaluated. In the present study, ophthalmologic toxicity was significantly more frequent in patients who experienced LVEF‐D. Although the mechanisms causing the toxicity of BRAF and MEKis are different between the eye and cardiomyocytes,^32^, ^36^, ^37^, ^38^, ^39^, ^40^, ^41^, ^42^, ^43^, ^44^ this association was expected as the majority of patients experienced retinal toxicities (central serous retinopathy and retinal pigment epithelial detachments) that related to MEK inhibition that is itself associated with LVEF‐D.^45^, ^46^, ^47^, ^48^, ^49^, ^50^ We could not determine herein whether ophthalmological toxicity occurs before or after LVEF‐D, but the significant association between these toxicities suggests that there should be a close ophthalmological monitoring when LVEF occurs, but also conversely.

The present study does, however, have certain limitations. First, its single‐center design is responsible for limited statistical power and possibly hinders the generalization of the results. Furthermore, the relatively small sample size and the small number of events could have led to an underestimation of some associations, such as a previous treatment with beta‐blockers and the absence of LVEF‐D. The retrospective nature of the study also limited led to a certain number of missing data, particularly concerning cardiovascular history, cardiovascular risk factors or previous cardiotropic treatments that may not have been recorded in medical files and be underestimated herein. Moreover, echocardiography data were not analyzed by a second blinded investigator.

In conclusion, LVEF‐D due to BRAF and MEKis appears fairly common but usually not severe, without impact on patient outcomes. The use of cardiotropic treatments, particularly beta‐blockers, seems to be beneficial, but their role in this context deserves to be confirmed by a larger prospective study. Cardio‐oncology units may be particularly useful for a better care of these patients with potential cardiotoxicity. The association between ophthalmological toxicity and LVEF‐D suggests a close ophthalmological monitoring when LVEF occurs, but also close cardiac monitoring when an ophthalmological toxicity is found.

## CONFLICT OF INTEREST

The authors declare they have non relevant conflict of interest.

## AUTHOR CONTRIBUTION

Mathilde Berger: conceptualization, formal analysis, investigation, writing – original draft, Mona Amini‐Adlé: conceptualization, formal analysis, investigation, review and editing, Delphine Maucort‐Boulch: statistical analyses, review; Philip Robinson: review and editing; Luc Thomas: resources, review and editing; Stéphane Dalle: resources, review and editing; Pierre‐Yves Courand: conceptualization, formal analysis, investigation, review and editing.

## Data Availability

I confirm that I have included a citation for available data in my references section, unless my article type is exempt.
