# Supplementary material for: Left ventricular ejection fraction decrease related to BRAF and/or MEK inhibitors in metastatic melanoma patients: A retrospective analysis
Source: Cancer Med. 2020 Feb 14;9(8):2611–20. doi: 10.1002/cam4.2922 (PMC7163110; doi:10.1002/cam4.2922)
Supplement: Supplementary file 1 [file CAM4-9-2611-s001.docx]

**Supplementary data**

There were 80 patients (90.9%) who experienced ≥ 1 extra-cardiovascular adverse event (AE) under treatment with BRAF and/or MEK inhibitors. The most frequent was elevated creatine phosphokinase (67.0%); this was found in 9 patients (75.0%) who experienced left ventricular ejection fraction (LVEF) decrease and in 50 patients (65.8%) who did not (p=0.74). There were 22 patients who experienced ophthalmological AEs, a majority of which were central serous retinopathy (13 patients). Ophthalmological AEs were significantly more frequent in patients who presented LVEF-D (50.0%) than those who did not (21.0%, p=0.006). A total of 19 patients presented ≥ 1 other cardiovascular AE events under treatment with BRAF and/or MEKis, including hypertension (4 patients), peripheral edema (4 patients), ischemic heart disease (3 patients), QT interval prolongation (3 patients), and pericardial effusion (3 patients). Among patients who presented significant LVEF-D, 1 patient experienced grade 2 hypertension, 1 patient grade 3 ischemic heart disease, and 1 patient grade 3 QT interval prolongation that led to the definitive discontinuation of the drug (Table 4).

Herein, 2 patients had a pericardial effusion under BRAF and/or MEKis. The first patient, treated with vemurafenib-cobimetinib, experienced an asymptomatic small pericardial effusion, fortuitously discovered on follow-up transthoracic echocardiography (TTE). There was no evidence of disease progression on computed tomography imaging. The effusion remained stable throughout the follow-up and did not require any specific intervention. The second patient was treated with vemurafenib alone. Three months after treatment initiation, he presented with acute dyspnea, chest pain, tachycardia, and jugular venous distension. TTE found a large circumferential pericardial effusion with pre-cardiac tamponade. Urgent pericardiocentesis was performed which drained 400 mL of serosanguinous pericardial fluid. Analysis of this fluid found a sterile exudate with no evidence of malignancy. There was no evidence of disease progression on computed tomography imaging. Evolution after drainage was favorable. Vemurafenib was discontinued for 10 days and resumed without recurrence of pericardial effusion afterwards.

Three patients experienced cardiac ischemia under treatment with BRAF and/or MEKis. The first patient was treated with BRAFi alone. She had no cardiovascular risk factor and no previous cardiovascular history. Four months after treatment initiation, follow-up electrocardiogram (ECG) found asymptomatic repolarization disorders with diffuse inverted T waves. TTE showed anterolateral hypokinesia with conserved LVEF at 60%, and troponins remained negative. Coronary angiography found a sub-occlusive stenosis of the left anterior descending artery treated with coronary angioplasty and stenting, with a favorable outcome. The second patient, who had a history of ischemic heart disease and type 2 diabetes, was treated with BRAFi alone. One year after treatment initiation, he presented with non-ST elevation myocardial infarction. ECG found inverted T waves in precordial leads, with elevated ultrasensitive troponins (600 ng/L, N < 14 ng/L). TTE found a LVEF-D (from 67% at baseline to 53%) with a lateral hypokinesia. Coronary angiography found a two vessels coronary artery disease, with tight stenosis of the left anterior descending artery and sub-occlusive stenosis of proximal left circumflex artery, requiring stent placement. LVEF did not recover afterwards. The third patient was treated with combination therapy. She had a history of chronic obliterative arterial disease of the lower limbs, hypertension, dyslipidemia, and an episode of transient ischemic attack one year ago. Eight months after treatment initiation, follow-up ECG found T waves inversion in lateral leads, with global hypokinesia and decrease in LVEF on TTE (from 67% at baseline to 38%). The patient was asymptomatic, and troponins remained negative. Coronary angiography found a significant stenosis (> 70%) of diagonal artery, requiring only medical treatment. Cardiac magnetic resonance imaging performed afterwards did not find any evidence of myocarditis. Evolution was favorable, and LVEF recovered in 4 months.

|  | Total  (n=88) | LVEF decrease  (n=12) | No LVEF decrease  (n=76) |
| --- | --- | --- | --- |
| Laboratory |  |  |  |
| Serum potassium (mmol/L) | 4.1 ± 0.4 | 4.2 ± 0.5 | 4.1 ± 0.4 |
| Serum calcium (mmol/L) | 2.4 ± 0.1 | 2.4 ± 0.2 | 2.4 ± 0.1 |
| Creatinine (µmol/L) | 76 ± 19 | 75 ± 14 | 76 ± 20 |
| eGFR – CKD-EPI (mL/min/1.73m^2^) | 91 ± 19 | 97 ± 18 | 90 ± 20 |
| eGFR – MDRD (mL/min/1.73m^2^) | 88 ± 21 | 95 ± 23 | 87 ± 21 |
| Hemoglobin (g/L) | 128 ± 28 | 128 ± 22 | 128 ± 29 |
| CPK (IU/L) | 78 ± 99 | 50 ± 38 | 82 ± 104 |
|  |  |  |  |
| Electrocardiogram |  |  |  |
| Heart rate (bpm) | 75 ± 13 | 72 ± 10 | 75 ± 14 |
| PR interval (ms) | 149 ± 26 | 143 ± 28 | 150 ± 26 |
| QRS duration (ms) | 90 ± 15 | 89 ± 16 | 92 ± 23 |
| 1^st^ degree atrioventricular block, n (%) | 2 (2.3) | 1 (8.3) | 1 (1.3) |
| Complete bundle branch block, n (%) | 4 (4.5) | 1 (8.3) | 3 (3.9) |
| Repolarization disorders, n (%) | 2 (2.3) | 1 (8.3) | 1 (1.3) |
| QT interval (ms) | 370 ± 32 | 385 ± 35 | 367 ± 31 |
| QTc (Bazett) (ms) | 410 ± 26 | 418 ± 25 | 408 ± 26 |
| QTc (Fridericia) (ms) | 395 ± 23 | 407 ± 26 | 394 ± 23 |
| Left ventricular hypertrophy, n (%) | 3 (3.4) | 0 | 3 (3.9) |
|  |  |  |  |
| Echocardiography |  |  |  |
| LVEF (%) | 65.6 ± 5.0 | 65.6 ± 5.0 | 65.7 ± 5.0 |
| Diastolic dysfunction, n (%) | 0 | 0 | 0 |
| Left atrium surface (cm^2^) | 15.5 ± 3.2 | 15.6 ± 2.8 | 15.5 ± 3.3 |
| PASP (mmHg) | 28.9 ± 6.3 | 29.5 ± 4.7 | 28.8 ± 6.5 |
| Significant valvulopathy, n (%) | 2 (2.3) | 0 | 2 (2.6) |
| Pericardial effusion, n (%) | 3 (3.4) | 1 (8.3) | 2 (2.6) |

Table S1 - Laboratory, ECG, and TTE characteristics of study population at baseline

*Unless otherwise stated, the data are given as means ± SD*

*eGFR, Estimated Glomerular Filtration Rate; CPK, Creatine phosphokinase; QTc, Corrected QT interval; LVEF, Left ventricular ejection fraction; PASP, pulmonary artery systolic pressure*

Table S2 - Variations of electrocardiographic and echocardiographic parameters during treatment with BRAF and/or MEK inhibitors for patients who did not experienced LVEF decrease

|  | Baseline | Final | p value |
| --- | --- | --- | --- |
| Electrocardiographic parameters | n=76 | n=73 |  |
| Heart rate (bpm) | 75 ± 14 | 77 ± 13 |  |
| PR interval (ms) | 150 ± 26 | 154 ± 26 |  |
| QRS duration (ms) | 92 ± 23 | 99 ± 30 |  |
| Repolarization disorders, n (%) | 1 (1.3) | 2 (2.6) |  |
| QT interval (ms) | 367 ± 31 | 366 ± 33 |  |
| QTc (Bazett) (ms) | 408 ± 26 | 411 ± 27 |  |
| QTc (Fridericia) (ms) | 394 ± 23 | 395 ± 25 |  |
| Cornell (mm) | 11.6 ± 5.1 | 11.1 ± 5.6 |  |
| RaVL (mm) | 4.7 ± 3.4 | 4.7 ± 3.1 |  |
|  |  |  |  |
| Echocardiographic parameters | n=76 | n=68 |  |
| LVEF (%) | 65.6 ± 5.0 | 64.4 ± 4.0 | 0.06 |
| E/A ratio | 1.1 ± 0.9 | 1.1 ± 0.6 |  |
| Deceleration time of E wave (ms) | 200 ± 55 | 215 ± 66 |  |
| E/E' ratio | 6.1 ± 1.9 | 6.6 ± 2.3 |  |
| Left atrium surface (cm^2^) | 15.5 ± 3.3 | 15.4 ± 3.3 |  |
| PASP (mmHg) | 28.8 ± 6.5 | 26.8 ± 5.0 |  |

*Unless otherwise stated, the data are given as means ± SD*

*QTc, Corrected QT interval; LVEF, Left ventricular ejection fraction; PASP, pulmonary artery systolic pressure*

Table S3: Other cardiovascular and extra-cardiovascular adverse events

|  | Total  (n=88) | LVEF decrease  (n=12) | No LVEF decrease  (n=76) | p value |
| --- | --- | --- | --- | --- |
| Other cardiovascular adverse events | 19 (21.6) | 3 (25.0) | 16 (21.1) |  |
| Hypertension | 4 (4.5) | 1 (8.3) | 3 (3.9) |  |
| Peripheric Edema | 4 (4.5) | 0 | 4 (5.3) |  |
| Ischemic heart disease | 3 (3.4) | 1 (8.3) | 2 (2.6) |  |
| Atrial fibrillation | 2 (2.3) | 0 | 2 (2.6) |  |
| Ventricular arrhythmias | 1 (1.1) | 0 | 1 (1.3) |  |
| ECG QT interval prolonged | 3 (3.4) | 1 (8.3) | 2 (2.6) |  |
| Pericardial effusion | 2 (2.3) | 0 | 2 (2.6) |  |
| Extra-cardiovascular adverse events | 80 (90.9) | 11 (91.7) | 69 (90.8) |  |
| Skin | 39 (44.3) | 7 (58.3) | 33 (42.7) |  |
| Eye | 22 (25.0) | 6 (50.0) | 16 (21.0) | 0.006 |
| Gastrointestinal | 34 (38.6) | 2 (16.7) | 32 (42.1) |  |
| Hepatobiliary | 12 (13.6) | 2 (16.7) | 10 (13.2) |  |
| Renal | 17 (19.3) | 2 (16.7) | 15 (19.7) |  |
| Respiratory, thoracic and----------- mediastinal | 5 (5.7) | 0 | 5 (6.6) |  |
| Nervous system | 3 (3.4) | 0 | 3 (3.9) |  |
| Musculoskeletal and -----------------connective tissue | 22 (25.0) | 4 (33.3) | 18 (23.6) |  |
| Blood and lymphatic system | 3 (3.4) | 0 | 3 (3.9) |  |
| General | 17 (19.3) | 2 (16.7) | 15 (19.7) |  |
| Ear and labyrinth | 1 (1.1) | 0 | 1 (1.3) |  |
| CPK increased | 59 (67.0) | 9 (75.0) | 50 (65.8) | 0.74 |

*Data are given as n (%).*

*CPK, Creatine phosphokinase*


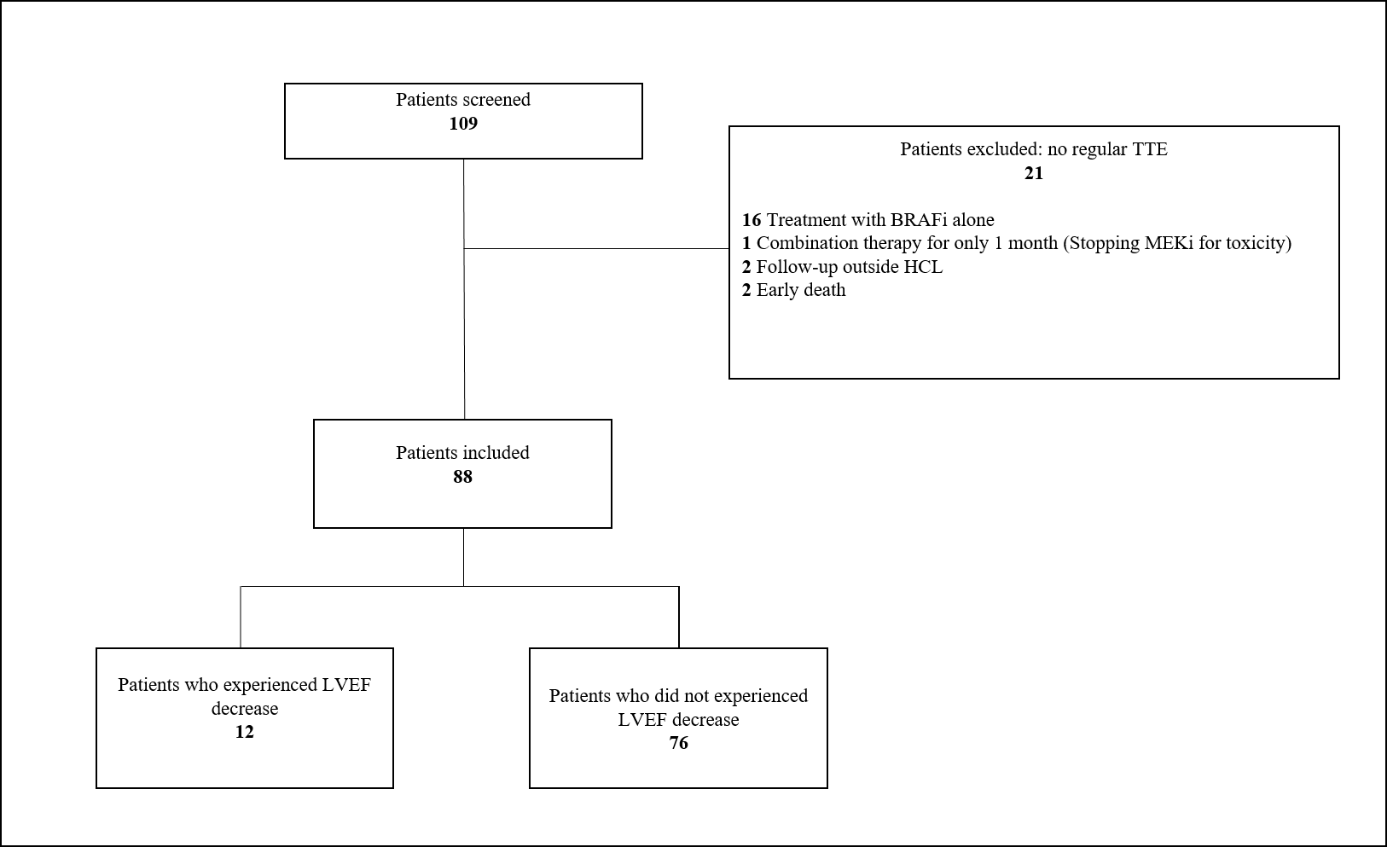


Figure S1. Study flowchart.

BRAFi, BRAF inhibitor; MEKi, MEK inhibitor; HCL, Hospices Civils de Lyon; LVEF, Left ventricular ejection fraction


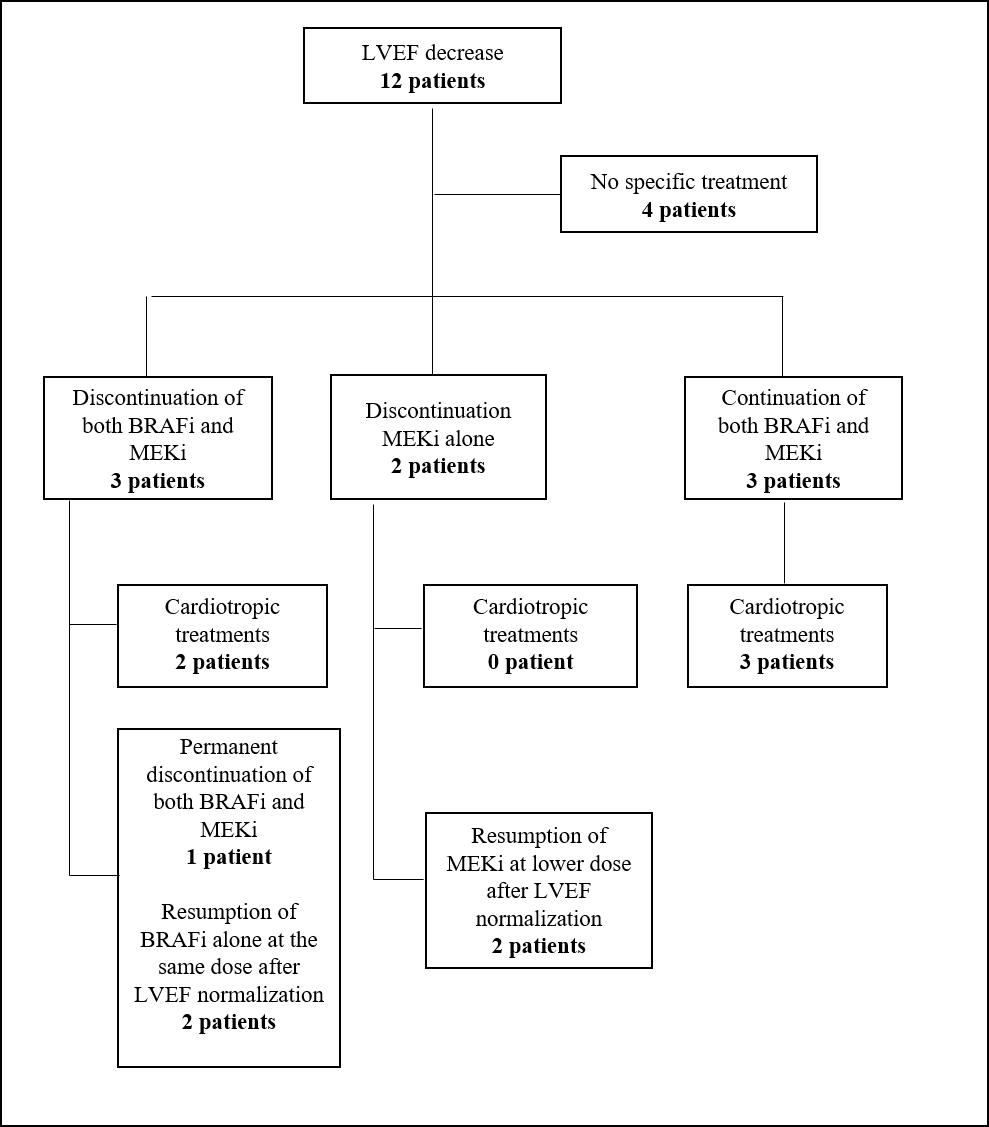


Figure S2. LVEF decrease management. BRAFi: BRAF inhibitor; MEKi: MEK inhibitor


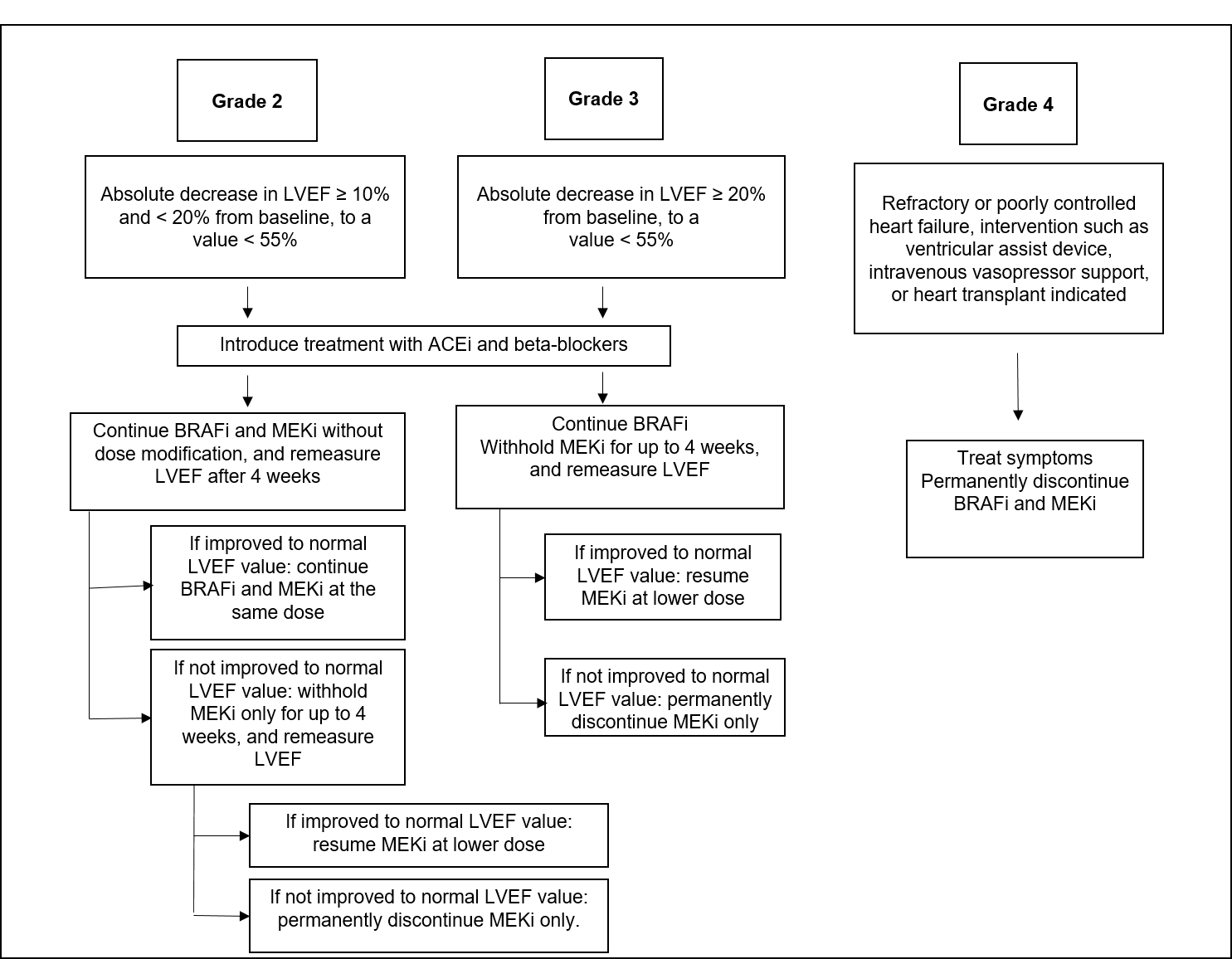


Figure S3. Suggested management algorithm of LVEF decrease according to CTCAE v5.0

LVEF, Left ventricular ejection fraction; ACEi, Angiotensin converting enzyme inhibitors; BRAFi, BRAF inhibitor; MEKi, MEK inhibitor.
